# PReS-FINAL-2193: Assessment of construct validity of new measures of global disease activity, physical function and quality of life in children with juvenile dermatomyositis

**DOI:** 10.1186/1546-0096-11-S2-O28

**Published:** 2013-12-05

**Authors:** C Ferrari, GC Varnier, A Consolaro, D Marafon, C Pilkington, S Maillard, M Jelusic-Drazic, A Civino, A Martini, A Ravelli

**Affiliations:** 1 Istituto Giannina Gaslini, Genoa, Italy; 2 Great Ormond Street Hospital, London, UK; 3 University Hospital, Zagreb, Croatia; 4 Az Ospedaliera C. Panico, Tricase (LE), Italy

## Introduction

Juvenile dermatomyositis (JDM) is a multisystem vasculopathic disease that affects primarily the skin and muscle and is characterized by high risk of morbidity and long-term damage. Regular patient assessment through standardized quantitative clinical measures is important to monitor the disease course over time and to evaluate treatment effectiveness. However, only a few outcome measures specifically validated for use in JDM are available.

## Objectives

To investigate the construct validity of the following new clinical measures for JDM developed by our group: 1) JDM-Act (global disease activity); 2) MyoFun (physical function); 3) Pediatric Rheumatology Quality of Life scale, PRQL (health related quality of life).

## Methods

Construct validity was assessed by computing the correlations between the new clinical measures and conventional JDM outcome measures by means of the Spearman's correlation coefficient. Correlations were considered good, moderate, or poor when the r_s _was > 0.7, 0.4-0.7, or < 0.4, respectively.

## Results

A total of 107 consecutive JDM patients (44 male, 63 female) seen in 4 pediatric rheumatology centres were included in the study. Mean disease duration was 3,1 years (IQR:1,0-5,8) and mean age at visit was 10 years (IQR:6,4-13,6). The table shows the Spearman's correlations of JDM-Act, MyoFun and PRQL scores with the values of conventional measures of disease activity and damage.

**Table 1:** 

	MMT	CMAS	DAS	CK	MDI	CHAQ	Parent global
JDM-Act Global VAS	-0.71	-0.72	0.86	0.31	0.26	0.59	0.66

MyoFun	-0.58	-0.56	0.51	0.38	0.12	0.83	0.79

PRQL-Physical Health	-0.52	-0.54	0.54	0.34	0.15	0.75	0.85

PRQL total score	-0.47	-0.48	0.46	0.27	0.14	0.71	0.82

## Conclusion

The new clinical measures showed good construct validity. By documenting this key measurement property, we have shown that the new tools are valid instruments for the assessment of children with JDM and are, therefore, potentially applicable in both clinical and research contexts. Because the new measures are simpler and shorter than most existing instruments, they may help foster the incorporation of quantitative clinical assessment in standard clinical practice.

## Disclosure of interest

None declared.

